# DNA methylation biomarkers in peripheral blood of patients with head and neck squamous cell carcinomas. A systematic review

**DOI:** 10.1371/journal.pone.0244101

**Published:** 2020-12-17

**Authors:** Christian Sander Danstrup, Mette Marcussen, Inge Søkilde Pedersen, Henrik Jacobsen, Karen Dybkær, Michael Gaihede

**Affiliations:** 1 Department of Otorhinolaryngology–Head & Neck Surgery and Audiology, Aalborg University Hospital, Aalborg, Denmark; 2 Clinical Cancer Research Center, Aalborg University Hospital, Aalborg, Denmark; 3 Department of Molecular Diagnostics, Aalborg University Hospital, Aalborg, Denmark; 4 Department of Clinical Medicine, Aalborg University, Aalborg, Denmark; 5 Department of Hematology, Aalborg University Hospital, Aalborg, Denmark; Johns Hopkins University, UNITED STATES

## Abstract

**Background:**

Head and neck squamous cell carcinomas (HNSCC) are often diagnosed in advanced stages. In search of new diagnostic tools, focus has shifted towards the biological properties of the HNSCC, and the number of different biomarkers under investigation is rapidly growing.

**Objectives:**

The objective was to review the current literature regarding aberrantly methylated DNA found in peripheral blood plasma or serum in patients with HNSCC and to evaluate the diagnostic accuracy of these changes.

**Methods:**

The inclusion criteria were clinical studies involving patients with verified HNSCC that reported findings of aberrantly methylated DNA in peripheral blood serum or plasma. We systematically searched PubMed, OVID Embase and Cochrane Library. In addition to the search, we performed forward and backward chaining in references and Web of Science. The protocol was registered in PROSPERO: CRD42019135406. Two authors independently extracted data. The quality and the risk of bias of the included studies were assessed by the QUADAS-2 tool.

**Results:**

A total of 1,743 studies were found eligible for screening, while ultimately seven studies were included. All studies were found to have methodological weaknesses, mainly concerning patient selection bias. The best individual marker of HNSCC was Septin 9 in plasma with a sensitivity of 57% and a specificity of 95%.

**Conclusions:**

None of the aberrantly methylated genes found in the retrieved studies are applicable as single diagnostic markers for HNSCC and the best gene-panels still lack diagnostic accuracy. Future studies may benefit from newer sequencing techniques but validation studies with well-designed cohorts are also needed in the process of developing epigenetic based diagnostic tests for HNSCC.

## Introduction

Head and neck cancer represents 4% of all malignancies with more than 650.000 new cases worldwide each year, where the majority of these cancers are squamous cell carcinomas (HNSCC) [1–3]. The overall 5 year survival of the patients is 60%, and it has improved only moderately over the last decades [4–6]. A major reason for this is probably that patients with HNSCC often present at later stages of their disease (III-IV as defined by the American Joint Committee on Cancer (AJCC) cancer staging manual [7]). Survival is inversely related to disease stage [8], and for patients with oro- and hypopharyngeal cancers, 72–75% present with late stages, with a 5 year survival rate below 50% [9, 10]. Smoking and alcohol consumption are major risk factors in HNSCC [11], however, the role of human papillomavirus (HPV) has within the last 10 years been increasingly recognized as a factor, particularly in the development of oropharyngeal cancer [12].The HPV-related oropharyngeal carcinomas have a better prognosis and show better response to treatment [13]. The differences between HPV-related and non HPV-related oropharyngeal carcinomas have caused that separate staging systems were established in the eighth edition of AJCC cancer staging manual [7].

In search of tools for earlier diagnosis, improved prognostic evaluation and detection of recurrent disease, focus has shifted towards the biological properties of HNSCC, and the number of biomarkers under investigation is rapidly growing. Initially, proteins and later changes in DNA, RNA (microRNA), and circulating tumor cells have been investigated. A particular evolving branch of DNA-research is epigenetic changes, where DNA-methylation represents one of the most studied modifications. This consists of the addition of a methyl (CH_3_) residue in a cytosine base proceeding a guanosine base, known as a CpG dinucleotide; in normal human DNA 3–6% of all cytosines are methylated [14].

Most CpG dinucleotides are found in CpG-rich regions, known as CpG islands [15]. 55–75% of human genes have been reported to have CpG rich promoters, and these are predominantly unmethylated in normal tissue [16, 17]. In the development of cancers, aberrant methylation of promoter-regions is of particular interest, as hypermethylation of tumor-suppressor genes may result in downregulation of the activity of the tumor suppressor function, while hypomethylation of promoter regions in oncogenes may result in an increased gene expression [18–21].

Cell-free DNA (cfDNA) from solid tumors has been demonstrated in the bloodstream. While the exact mechanism behind such release of cfDNA is still debated, apoptotic and necrotic cells are thought to be the origin [22, 23]. If these DNA-fragments can be detected and their methylation-status determined, this holds the potential of becoming an important tool in early demonstration and diagnosis of malignant solid tumors. In several types of malignant solid tumors, but not HNSCC, commercial tests based on aberrant methylation are already available (e.g. the Epi proColon^®^ and Cologuard^®^ for colorectal-cancer screening and the Epi proLung^®^ in detection of lung cancer) [24–29].

Based on this background, the primary objective of this paper was to review the current literature of the usage of aberrantly methylated DNA found in the peripheral blood plasma or serum of patients with HNSCC and evaluate the diagnostic accuracy of these changes. A second objective was to evaluate the potential prognostic value of such findings.

## Materials and methods

We conducted a systematic review according to the Preferred Reporting Items for Systematic review and Meta-analysis (PRISMA) guidelines (S1 Checklist) [30]. The protocol was registered in the PROSPERO database for systematic reviews with registration number: CRD42019135406 [31].

### Inclusion criteria

Clinical studies involving patients with histopathologically verified HNSCC that reported findings of aberrantly methylated DNA in peripheral blood serum or plasma were eligible.

### Exclusion criteria

Studies were excluded, if they were: (1) performed in animals; (2) only describing aberrantly methylated DNA in solid tumors; (3) published only as conference abstracts; (4) published only in a review; or (5) published in a language other than English.

### Search strategy

In June 2019, we performed a systematic literature search in OVID Embase, PubMed and Cochrane Library. No limits were placed on the publication dates in our search. In addition to the search, we performed forward and backward chaining in references and Web of Science. The search strategy was structured by one reviewer (C.D.) in collaboration with Pernille Gaardsted, research librarian at the Medical Library, Aalborg University Hospital. An updated search was performed in March 2020. The full search strategy appears in S1 Table.

### Study selection

**Fig 1:**
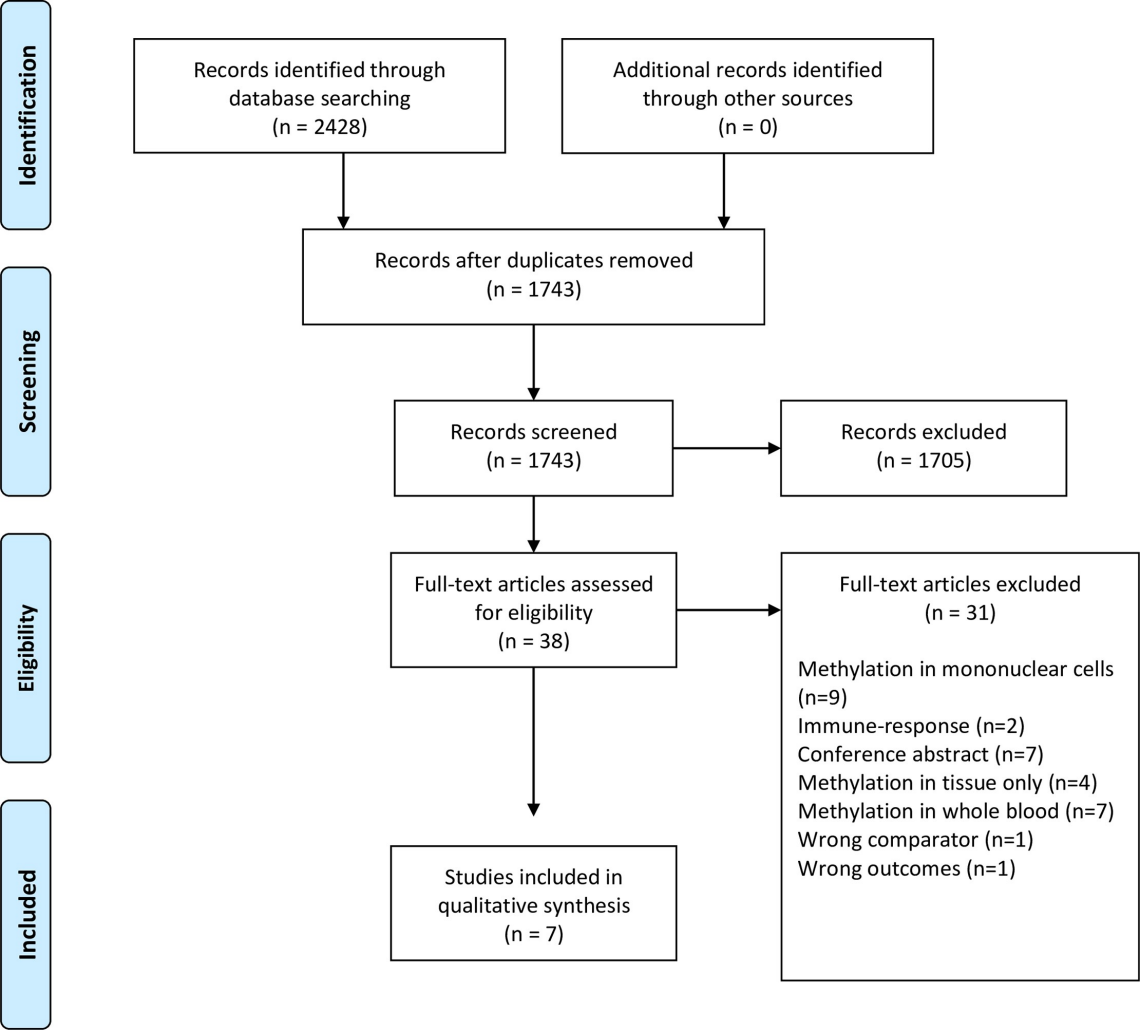
PRISMA flow diagram. From Moher et al. [30].

Papers found according to our search strategy were imported into the Covidence online software (Covidence, Melbourne AUS for data management and literature screening [32]). After duplicate removal, one reviewer (C.D.) screened titles and abstracts of all potential studies using the inclusion criteria. Then all full text articles were assessed for eligibility by two independent reviewers (C.D., M.M.). Any disagreement was resolved by consensus. A flowchart showing the study selection process in according to PRISMA was generated (Fig 1).

### Data collection process

Two authors (C.D., M.M.) independently extracted data and assessed the risk of bias in the included studies. Data extraction included the name of first author, year of publication, number of HNSCC cases, number of controls, medium examined (plasma/serum), type of methylation analysis used, diagnostic performance, prognostic value of the methylation status in HNSCC patients measured by recurrence free survival and overall survival. Any disagreement was resolved by consensus.

Due to the heterogenicity of the included studies a meta-analysis was not considered meaningful and a structured narrative synthesis was performed.

### Quality assessment and assessment of the risk of bias

The quality and the risk of bias of the included studies were assessed by the QUADAS-2 tool [33]. The tool is designed to assess the quality of primary diagnostic accuracy studies. The following domains were evaluated: Patient selection, index test, reference standard, flow and timing.

## Results

A total of 2428 studies were imported into Covidence. After removal of duplicates by Covidence (685) a total of 1743 studies were eligible for screening according to the inclusion criteria. The screening process resulted in the removal of 1705 papers (incl. two duplicates found during the screening) and left 38 papers for assessment. The full text assessment resulted in the further exclusion of 31 studies and the inclusion of a total of 7 studies for final analysis [34–40] (Fig 1). The reference lists of the included articles were screened for additional relevant articles, but none met the inclusion criteria.

The earliest study was performed in 2000 [40], and the latest studies performed in 2017 [37, 38]. Six of the seven studies used gene panels based on results derived from earlier published studies of HNSCC or other malignant tumors (e.g. lung, breast and colorectal cancers), while Mydlarz et al. chose a panel based on own earlier findings in saliva [35]. All studies evaluated the methylation status of the primary tumor and of peripheral blood, serum, or plasma with focus on a limited number of genes. None of the investigators performed a broad or genome-wide analysis. The studies show a large variation in included number of cases and controls. Thus, 425 HNSCC patients were included in the study by Schröck et al. [37], contrasting the 17 patients with oral cancer described by Nakahara et al. [39].

It is noteworthy that studies [37, 38] are from the same research group and that studies [34, 35, 40] are also from one group.

### Results from studies of methylation in serum

In 2008, Carvalho et al. [34] published a study on evaluation of promoter hypermethylation in body fluids as a diagnostic tool in patients with HNSCC and healthy controls using quantitative methylation-specific polymerase chain reaction (qMSP). They examined a panel of 21 genes that previously were described as hypermethylated in HNSCC or other solid tumors. Six genes found in serum were significantly associated with HNSCC. Hypermethylation of each gene was treated as a binary variable (methylation versus no methylation) and each gene was evaluated separately and combined in a panel of the best possible combinations regarding sensitivity and specificity (Table 1).

**Table 1:** Diagnostic value of DNA methylation in plasma or serum.

Gene	Name	Cases/controls	Concordance rate between methylation in tumor and blood	Anatomical site	Stage	Specimen	Sensitivity	Specificity	AUC	Method	Reference
CDH1	Cadherin 1	62/320		All	NA	Serum	32%	73%	0.53	qMSP^b^	Carvalho et al. [34] (2008)
CCND2	Cyclin D2	47/284		All	NA	Serum	6%	98%	0.52	qMSP^b^	Carvalho et al. [34] (2008)
TGFBR2	Transforming Growth Factor Beta Receptor 2	37/134		All	NA	Serum	8%	94%	0.51	qMSP^b^	Carvalho et al. [34] (2008)
TIMP3	TIMP Metallopeptidase Inhibitor 3	50/296		All	NA	Serum	10%	95%	0.52	qMSP^b^	Carvalho et al. [34] (2008)
HIC1	Hypermethylated in Cancer 1	70/373		All	NA	Serum	31%	93%	0.62	qMSP^b^	Carvalho et al. [34] (2008)
PGP9.5	Neuron Cytoplasmatic Protein 9.5	52/203		All	NA	Serum	8%	98%	0.53	qMSP^b^	Carvalho et al. [34] (2008)
SEPT9^1^	Septin 9	137/170	-	All	All	Plasma	62%	92%	0.79	quasi-digital PCR	De Vos et al. [38] (2017)
SHOX2^1^	Short Stature Homeobox 2	137/170	-	All	All	Plasma	53%	87%	0.78	quasi-digital PCR	De Vos et al. [38] (2017)
SEPT9^2^	Septin 9	141/102	-	All	All	Plasma	55%	90%	0.75	quasi-digital PCR	De Vos et al. [38] (2017)
SHOX2^2^	Short Stature Homeobox 2	141/102	-	All	All	Plasma	43%	95%	0.77	quasi-digital PCR	De Vos et al. [38] (2017)
EDNRB	Endothelin Receptor Type B	100/50	-	All	All	Serum	10%	100%		qMSP^b^	Mydlarz et al. [35] (2016)
DCC	Deleted in Colo-rectal Cancer	100/50	-	All	All	Serum	2%	100%		qMSP^b^	Mydlarz et al. [35] (2016)
CDKN2A	Cyklin-Dependent Kinase Inhibitor 2A	100/50	-	All	All	Serum	1%	100%		qMSP^b^	Mydlarz et al. [35] (2016)
CDKN2A	Cyklin-Dependent Kinase Inhibitor 2A	17/8	65%	Oral	All	Serum	35%	100%		MSP^a^	Nakahara et al. [39] (2005)
CDKN2A	Cyklin-Dependent Kinase Inhibitor 2A	50/0	27%	All	All	Serum	4%	-		MSP^a^	Sanchez-Cespedes et al. [40] (2000)
MGMT	O^6^-Methylguanine-DNA-Methyltransferase	50/0	33%	All	All	Serum	7%	-		MSP^a^	Sanchez-Cespedes et al. [40] (2000)
DAPK	Death-Associated Protein Kinase	50/0	18%	All	All	Serum	2%	-		MSP^a^	Sanchez-Cespedes et al. [40] (2000)
GSTP1	Glutathione S-transferase p1	50/0	0%	All	All	Serum	0%	-		MSP^a^	Sanchez-Cespedes et al. [40] (2000)
SHOX2^1^	Short Stature Homeobox 2	137/122	-	All	All	Plasma	50%	95%	0.80	qPCR	Schröck et al. [37] (2017)
SEPT9^1^	Septin 9	137/122	-	All	All	Plasma	57%	95%	0.79	qPCR	Schröck et al. [37] (2017)
SHOX2^2^	Short Stature Homeobox 2	141/102	-	All	All	Plasma			0.79	qPCR	Schröck et al. [37] (2017)
SEPT9^2^	Septin 9	141/102	-	All	All	Plasma			0.74	qPCR	Schröck et al. [37] (2017)
CDKN2A	Cyklin-Dependent Kinase Inhibitor 2A	20/24	49%	All	All	Plasma	65% (13/20)	67% (16/24)		Real time PCR	Wong et al. [36] (2003)
CDKN2B	Cyklin-Dependent Kinase Inhibitor 2B	20/24	60%	All	All	Plasma	60% (12/20)	50% (12/24)		Real time PCR	Wong et al. [36] (2003)

a: Methylation specific PCR.

b: Quantitative methylation specific PCR.

c: Combined bisulfite restriction analysis.

1: Training Cohort.

2: Testing cohort.

*The test is between OSCC and OP. No healthy controls.

The performances of the selected panels were explored using multivariable logistic regression and receiver operating characteristic (ROC) curves. Internal validation of the logistic regression was done by using an approximation to the leave-one-out jackknife procedure. Of the six genes only one, Hypermethylated in Cancer 1 (HIC1), was considered useful as a diagnostic single-gene-marker with a sensitivity of 31.4% and a specificity of 92.5%. No combinations of the selected genes were able to surpass the specificity of HIC1. A panel of five genes showed a sensitivity of 87.2% and a specificity of 42.3%.

In their study, Mydlarz et al. [35] evaluated 100 HNSCC patients and 50 controls using qMSP to detect hypermethylated Endothelin Receptor Type B (EDNRB), Deleted in Colorectal Cancer (DCC) and Cyclin-Dependent Kinase Inhibitor 2A (CDKN2A). Detection of hypermethylation was interpreted in a binary fashion without any cut-off as no methylation was found in normal serum. In the HNSCC group, genes were hypermethylated in 10%, 2% and 1%, respectively. No hypermethylation of the specific genes could be demonstrated in the control group. There was no significant association between serum positivity for hypermethylated genes and local recurrence free and overall survival (Table 2).

**Table 2:** Prognostic value of DNA methylation in plasma or serum.

Gene(s)	Name	Anatomical site	Stage	Local recurrence-free survival (hazard ratio)	Overall survival (hazard ratio)	Method	Reference
EDNRB/DCC/CDKN2A		All	All	1.35^c^ (0.17–10.5)	1.35^c^ (0.41–4.43)	qMSP	Mydlarz et al. [35]
SEPT9^1^	Septin 9	All	All	-	5.27^a^ (2.03–13.68)	qPCR	Schröck et al. [37]
SHOX2^1^	Short statue Homeobox 2	All	All	-	2.32^a^ (1.12–4.83)	qPCR	Schröck et al. [37]
SEPT9^1^	Septin 9	All	All	1.32^b^ (1.09–1.59)	1.23^b^ (1.07–1.41)	qPCR	Schröck et al. [37]
SEPT9^2^	Septin 9	All	All	-	2.78^a^(1.16–6.67)	qPCR	Schröck et al. [37]
SHOX2^2^	Short statue Homeobox 2	All	All	-	2.50^a^ (1.12–5.60)	qPCR	Schröck et al. [37]
SEPT9^1^	Septin 9	All	All	-	1.90^d^ (1.43–2.69)	quasi-digital PCR	De Vos et al. [38]
SHOX2^1^	Short statue Homeobox 2	All	All	-	1.85^d^ (1.18–2.91)	quasi-digital PCR	De Vos et al. [38]
SEPT9^2^	Septin 9	All	All	-	1.67^d^ (1.13–2.45)	quasi-digital PCR	De Vos et al. [38]
SHOX2^2^	Short statue Homeobox 2	All	All	-	1.71^d^ (1.05–2.80)	quasi-digital PCR	De Vos et al. [38]

a: Univariate cox proportional hazards analysis of the 129 cases with positive serum samples using the introduced cut-off value.

b: Multivariate cox proportional hazards analysis of the 129 cases using the DNA methylations levels as a continuous variable.

c: Univariate proportional hazards analysis.

d: Univariate cox proportional hazards analysis.

1: Training Cohort.

2: Testing Cohort.

In another study, Nakahara et al. [39] examined hypermethylation of CDKN2A in biopsies and serum of 17 oral squamous cell carcinoma (OSCC) patients and eight healthy controls using MSP. Methylation was treated as a binary outcome. They found a sensitivity of 35% and a specificity of 100% in serum. The authors performed pre- and post-treatment serum analyses, and all patients with aberrant methylation in serum before treatment were non-methylated at two months post-treatment follow up. Four patients had recurrence and three of these patients, had aberrant methylation in the peripheral blood. In addition, all three had aberrant methylation in both tumor and serum prior to treatment.

Using MSP for either methylated or unmethylated sequences, Sanchez-Cespedes et al. [40] published a study in 2000 examining aberrant methylation of four genes, CDKN2A, 0^6^-methylguanine-DNA-methyltransferase (MGMT), Death-associated protein kinase (DAPK), Glutathione S-transferase P1 (GSTP1). Out of 95 HNSCC tumor and serum samples, 52 patients had aberrant methylation in the primary tumor. MSP was unsuccessful in two cases and therefore only 50 serum samples were evaluated. Twenty-one of 50 patients had the same methylation changes in the serum as in the primary tumor. CDKN2A was positive in eight serum samples, MGMT in 14 and DAPK in three. GSTP1 hypermethylation was not found in any tumor or serum sample. As control subjects, they analyzed serum of 25 patients, who did not have aberrant methylation in the primary tumor. None of the serum samples in the control group, showed aberrant methylation of the four genes. Seven of the patients had a new serum sample post-treatment. None of the methylation-negative patients had converted, however, one DAPK-positive patient had become negative, whereas two patients still were methylation-positive 5–6 months post-treatment.

### Results from studies of methylation in plasma

Short Stature Homeobox 2 (SHOX2) and Septin 9 (SEPT9) have been examined by Schröck et al. and De Vos et al. [37, 38]. First, a *training cohort* with 284 patients with HNSCC and 122 matched healthy controls and a *test cohort* with 141 patients with HNSCC and 102 matched healthy individuals were tested for these two biomarkers with triplex quantitative PCR. Only 137 of 284 patients with HNSCC in the *training cohort*, had baseline blood plasma samples available. The authors introduced a methylation cut-off value to dichotomize the methylation values and levels below these values were considered sporadic background methylation. Matched tumor samples and pretherapeutic methylation in plasma from 55 patients were analyzed [37]. SHOX2 methylation in plasma correlated significantly with tissue levels (Spearman’s p = 0.36, P = 0,007), while SEPT9 showed a suggestive trend (Spearman’s p = 0.25, P = 0.067). ROC curves have been computed as a measure of diagnostic accuracy and in the *training cohort* SHOX2/SEPT9 sensitivity and specificity were reported to be 59% and 96%, respectively (Table 1). Using univariate Cox proportional hazard analysis for both cohorts, the authors found a significantly higher risk of death for methylation positive patients for both markers [37, 38] (Table 2).

In their second study [38], the team evaluated three different algorithms to asses methylated cfDNA; Relative quantification, Absolute quantification and Quasi-Digital PCR. They used the same two markers (SEPT9 and SHOX2) and almost the same training and testing cohort as in their previous study [37], only adding 48 extra controls to the *training cohort*. All three algorithms showed similar Area Under the Curve (AUC) in the ROC curves (Performance for Quasi-Digital PCR can be found in Table 1). In prognostic evaluation in both cohorts, all three algorithms also showed significantly increased hazard ratios for methylation positive patients (Performance for Quasi-Digital PCR can be found in Table 2).

Finally, Wong and colleagues [36], used real time PCR to examine promoter methylation of the p16 and p15 genes in plasma. Methylated p16 was found in 65% of the patients with HNSCC in contrast to 20% of the healthy controls. Methylated p15 was seen in the plasma of 60% of the patients with HNSCC and in 50% of the healthy controls. The mean concentration of methylated p16 and p15 was significantly higher in HNSCC patients compared to healthy controls (p = 0.016 and 0.0037 respectively), but no cut-off values for background methylation was defined.

### Risk of bias

The QUADAS-2 evaluation of the included studies showed that all studies had a high or unclear risk of bias related to patient selection. None of the studies explicitly described how patients or controls were enrolled, e.g. consecutively or randomly or other criteria. Furthermore, all the studies were case control by design. All index-tests were interpreted with the knowledge of the reference standard in the HNSCC groups. None of the included studies described the precise flow and timing regarding time from diagnosis to biopsy and blood sampling. (An overview of the QUADAS-2 evaluation appears in Table 3).

**Table 3:** Risk of bias and applicability concerns.

Study	RISK OF BIAS				APPLICABILITY CONCERNS		
	PATIENT SELECTION	INDEX TEST	REFERENCE STANDARD	FLOW AND TIMING	PATIENT SELECTION	INDEX TEST	REFERENCE STANDARD
Carvalho et al. [34]	High	High	Low	Unclear	Low	Low	Low
Mydlarz et al. [35]	Unclear	High	Low	Unclear	Low	Low	Low
Nakahara et al. [39]	High	High	Low	Unclear	Unclear	Low	Low
Sanchez-Cespedes et al. [40]	Unclear	High	Low	Unclear	High	Unclear	Low
Schröck et al. [37]	High	High	Low	Unclear	Low	Low	Low
De Vos et al. [38]	High	High	Low	Unclear	Low	Low	Low
Wong et al. [36]	High	High	Low	Unclear	Unclear	Low	Low

### Applicability concerns

There were no concerns regarding the condition as defined by the reference standard in any of the included studies. One study [40], was graded as unclear concerning the index test, as no healthy control group was included.

Two studies [27, 30], did not report any information on the control groups, and they were judged as unclear regarding patient selection. As no healthy control group was defined in the study by Sanchez-Cespedes et al. [40], the concern regarding patient selection was high. The remaining four studies [34, 35, 37, 38], were judged as low as they had matched control groups and provided tables with demographic information regarding age, gender, tobacco- and alcohol consumption. (An overview of the applicability concerns is given in Table 3)

## Discussion

Epigenetic changes, including aberrant DNA methylation, in human cancers was initially described more than thirty years ago [41]. With the development of new laboratory techniques and assays, the ability to examine body fluids, especially blood, for circulating tumor cells or tumor DNA has inspired the search of new diagnostic and prognostic tools in cancer. The objective of this review was to provide an overview of aberrantly methylated DNA found in plasma or serum of peripheral blood and their diagnostic and prognostic potential in patients with HNSCC.

The majority of the studies, we retrieved in this systematic review, described both sensitivity and specificity of the different aberrantly methylated genes, whereas the study by Sanchez-Cespedes et al. [40] did not include a healthy control group, so that specificity could not be calculated.

Not all included studies presented both diagnostic and prognostic data, as only three studies [35, 37, 38], described follow-up, and therefore, they were able to calculate hazard ratios on local recurrence free survival and overall survival.

Both single genes and gene-panels were evaluated. The best individual marker was found by Schröck et al. [37] as detected hypermethylated SEPT9 in plasma had a 57% sensitivity and 95% specificity. Hypermethylated SEPT9 was also assessed in studies of other solid tumors, e.g. the Epi proColon^®^ that is a commercially available blood-based DNA hypermethylation test screening for colorectal cancer. As a single marker, the assay reached a sensitivity of 48.2% and specificity of 91.5% in a large prospective study with 6874 asymptomatic patients scheduled for colorectal cancer screening [42].

When combining both SHOX2 and SEPT9 (Mean_Shox2/SEPT9_), Schröck et al. [37] demonstrated that the sensitivity and specificity can be improved to 59% and 96%, respectively. In the study by Carvalho et al. [34] the best combined gene panel generated a sensitivity of 87% and a specificity of 42%. Their study was limited by the fact that not all genes were analyzed in all HNSCC- and control samples.

### Anatomical subsite, stage and HPV status

The anatomical subsite and HNSCC tumor stage may also play a role in the release of tumor DNA, and hence, the methylation-findings in peripheral blood. Thus, tumors in subsites with rich lymphatic drainage and blood supply may possibly be detected earlier. For example, the lymphatic drainage and blood supply of the larynx is remarkably different to other subsites due to the dual embryological origin [43]. Tumor size may also play a role, as small T1 tumors of the larynx will present a small cell-turnover compared to a large invasive T4b supraglottic tumor invading the prevertebral space.

The incidence of HPV-related HNSCC has been increasing during the last decades and is now described as an independent subtype with a distinct morphological profile and occurs primarily in the oropharynx of younger patients without tobacco and alcohol as co-factors [44, 45]. It has been shown that expression of the p16 protein is highly correlated to HPV in oropharyngeal cancer where the protein is upregulated in contrast to non HPV-related HNSCC. Due to this strong correlation, immunohistochemical analysis of p16 are used as a surrogate marker of HPV-related HNSCC [46].

This etiological linkage and role of HPV should be taken into consideration when evaluating methylation profiles as these may differ between HPV-related and non HPV-related HNSCC. This has been addressed by Delgi Esposti et al. [47]. Using pooled genome-wide analyses they found that HPV infection affects the DNA methylation in HNSCC across different anatomical subsites.

Of the seven studies included, only Mydlarz et al. [35] reported the HPV-status in their HNSCC patients, but did not mention the method used to determine HPV-status. They described the HPV- status related to survival but not in relation to the biomarkers examined. Five studies [34–36, 39, 40] evaluated methylation of the p16 promoter, but none described or discussed the findings in relation to HPV and potential differences between HPV-related and non HPV-related HNSCC.

The mechanism of DNA release into the circulation it not fully clarified. However, it is suggested that circulating DNA is derived from apoptotic or neoplastic cells or from tumor cells that are not capable of generating metastases [22, 23, 48]. Carvalho et al. [34] did not report the tumor size and stage of the cancer patients examined, while Wong et al. [36] only reported methylation status of the primary tumor in relation to tumor size. All positive samples in the study by Mydlarz et al. [35] were Stage IV cancers and Schröck et al. [37] found a strong correlation between the level of the biomarkers in plasma and tumor- and nodal-category.

This should be taken into consideration when evaluating blood-based biomarkers as screening tools for HNSCC and if possible, anatomical subsite, HPV status, tumor size and stage should be reported when evaluating the potential diagnostic and prognostic biomarkers.

### Examination of plasma versus serum–flow and timing

The use of plasma and serum was almost equally distributed as four studies [34, 35, 39, 40], evaluated methylation in serum, and three studies [36–38] evaluated methylation in plasma.

In general, the amount of cell free DNA is higher in serum compared to plasma (2–24 times). Therefore, serum is suggested as the best source for evaluation, however, Jung et al. [49] examined the changes in the concentration of DNA in serum and plasma during storage and handling of blood samples. They found no change in DNA concentration in plasma stored 8h at room temperature, but time delay and storage temperature had significant impact (3.8–4.8 times higher for samples stored 2-8h at room temperature) on the concentration of DNA in serum.

Of the included studies, only Schröck et al. [37] and De Vos et al. [38] explicitly described the plasma preparation regarding timing, as plasma were prepared within 2h after sampling.

None of the included studies described the flow and timing between the biopsy (histologically verifying the diagnosis) and the blood samples. If a large biopsy in a vascularized field is taken, it may cause a release of tumor-cells in the bloodstream, and hence, an elevation of cfDNA in the blood sample afterwards. Henriksen at al. demonstrated that total cfDNA levels were elevated up to four weeks after surgery in patients undergoing colorectal cancer surgery [50]. Therefore, when studying biomarkers potentially released from tumor tissue, the study should preferable include a measurement the DNA methylation status before (and after) the primary biopsy with documented flow and timing, or at least describe the flow of sample collection.

### Cut-off values and background methylation

Only two studies [35, 39] did not find any aberrant methylation of the evaluated biomarkers in the healthy control groups. The fact that the other studies find aberrant methylation in healthy controls suggest that other factors may contribute to the methylation-level in general. This issue was only addressed by Schröck and others [37] and defined as sporadic background methylation. Therefore, a methylation level cutoff was introduced.

Wong et al. [36] evaluated the resection-margins of 29 smoking patients with HNSCC. They found that methylation-levels of the p15 promoter were significantly higher in resection margins (and histopathologic healthy tissue) in chronic smokers compared to non-smokers. These findings have also been documented by others [51, 52]. When evaluating a biomarker as a screening or diagnostic tool, it should be evaluated in a group of people at risk. Matched controls regarding age, gender, use of tobacco, alcohol and general health-status should be preferred. Of the included studies, two failed to report demographics of their control patients, hence introducing potential bias in the patient selection [36, 39].

### Reflections of tumor-methylation in peripheral blood–concordance rate

All the included studies describe that methylation analyses of the primary tumors have been performed, but not all described the concordance between tumor- and the serum-methylation.

Using Spearman correlation, Schröck et al. analyzed 55 matched tumor and blood samples and found a significant correlation for SHOX2 but not for SEPT9. Sanchez-Cespedes et al. [40] found that 42% of the examined patients had the same methylation changes in serum and the primary tumor.

In the study of aberrantly methylated CDKN2A by Nakahara et al. [39], 54,5% of the patients had matching tumor and serum changes. In a study of CDKN2A in 94 colorectal cancer patients, a concordance rate of 30% was found [53], and in a study of primary lung cancer (using MSP) by Ooki et al., the concordance rate was between 20–56% in a six-gene panel [54].

None of the included studies had a perfect concordance rate of methylation in tumor and plasma/ serum, which raises the question, why that is so. HNSCC are highly heterogeneous tumors and different methylation-profiles may exist even within the tumor. Thus, different biopsy-sites may yield different methylation-profiles. If different methylation-profiles exists, this may also be reflected in the DNA released to the circulation.

### Prognostic value of aberrant blood methylation in HNSCC and post-treatment surveillance

Several studies have evaluated the prognostic value of aberrant methylation profiles in the primary tumors of HNSCC [55–59]. Of the included studies, only three [35, 37, 38] had follow-up, thus describing local recurrence free- and overall survival. Mydlarz et al. [35] did not find a significant association between local recurrence or overall survival and promoter hypermethylation of the three genes examined (Table 2).

Schröck et al. [37] performed univariate proportional hazards analyses on both cohorts and multivariate analyses on their *training cohort*. In the univariate analyses, patients with positive plasma levels of either SEPT9 or SHOX2 were at higher risk of death compared to the methylation negative patients. In multivariate analyses of the 129 patients in their *training cohort*, SEPT9 proved to be an independent prognostic factor regarding local recurrence free survival (HR: 1.32 CI:1.09–1.59) and overall survival (HR:1.23 CI: 1.07–1.41) (Table 2). De Vos et al. [38] found both SEPT9 and SHOX2 to be significant prognostic factors regarding overall survival in all tested quantification algorithms (Table 2).

Post-treatment evaluation of aberrant methylated DNA was only reported in two of the seven studies included [39, 40]. Nakahara et al. found that all six patients with positive tests with regard to pre-treatment aberrant methylated DNA had converted to negative tests two months after treatment; four of these patients were later diagnosed with recurrent disease, and three had converted back to positive tests [39]. Sanchez-Cespedes et al. reported from post-treatment samples that in three patients with positive pre-treatment tests, only one patient became negative after treatment, while in two patients’ tests were still positive at five to six months of follow-up. It was not reported, whether these patients later had recurrent disease [40]. The role of post-treatment profiles of aberrant methylated DNA in HNSCC remains unclear. Symonds et al. reported conversion of positive to negative tests in 35 of 47 patients with colorectal-cancer [60]. Thus, it seems relevant to pursue this aspect in patients with HNSCC as it may reveal an important method for surveillance.

### Methods used in methylation analyses

The included studies use different methods in their methylation analyses. Methylation-specific PCR (MSP) was one of the earliest methods used in methylation studies and was initially described by Herman et al. in 1996 [61]. The method uses primers to recognize methylated- and unmethylated CpG’s. The method is cost-effective, but has a disadvantage of false positives results [62]. MSP have been used in two studies in this review, which are also two of the oldest studies (Sanchez-Cespedes et al. in 2000 and Nakahara et al. in 2005).

Of more recent methods, Yokoi et al. named the quantitative methylation specific PCR (qMSP) as the “gold standard” of methylation analyses in a review from 2017 [63]. qMSP has been used in another two of the included studies [34, 35]. The group with Schröck and De Vos used triplex quantitative PCR (qPCR) and Wong et al. used real time PCR [36].

There are several challenges in the detection and quantification of cfDNA. The amount of circulating cfDNA from tumor is generally very limited and have a short half-life. Therefore, the methods used are particularly susceptible to timing and workflow, and optimal standardized methods are needed in order to obtain a high performance.

All the included studies used targeted assays covering only a few numbers of known promoters. Recent technological progress has enabled whole genome methylation analyses e.g. Methylated DNA immunoprecipitation sequencing (MeDIPseq), whole-genome bisulfite sequencing or the Infinium MethylationEpic Array [64, 65]. Each method has its advantages but common for all are the genome wide coverage compared to targeted assays.

Using comprehensive integrative molecular analyses of 10.000 specimens of primary tumors across 33 types of cancer in The Cancer Genome Atlas, Hoadley et al. [66] shoved that the influence of cell type was evident in DNA-methylation based clustering. Using MeDIP-seq, Shen et al. [67] demonstrated detection of early-stage pancreatic cancer trough cfDNA in plasma but also the potential of multi-cancer classification using the same protocol in a cohort of 189 plasma samples from 7 different tumor types. Early-stage detection across >50 different cancers, including HNSCC by analyzing methylation signatures in cfDNA with advanced computational biology has also recently been described by Liu et al. [68].

Ideally, each subtype of cancer presents a unique, detectable cfDNA signature and a genome-wide analysis with improved bioinformatics would be able to detect the cancer present at an early stage and the cell or tissue of origin.

### Methods used in bisulfite conversion

Despite using different PCR-methods, all the included studies use bisulfite treatment of purified DNA. Efficient bisulfite conversion of DNA prior to PCR or any other methylation analysis is an important step. Incomplete conversion may result in overestimation of methylation. Furthermore, bisulfite treatment may result in degradation and loss of DNA, especially when using samples of less than 200 ng DNA. In these cases, more than 95% of the bisulfite-treated DNA is lost, if using standard procedures [69]. However high recovery can be achieved through a rapid bisulfite-treatment protocol [70]. The challenge regarding efficient bisulfite conversion has also been addressed by Ørntoft and colleagues [71]. They compared 12 different kits for bisulfite conversion of circulating cfDNA and found that mean recovery ranged between 9 and 32% and a bisulfite conversion efficiency of 97–99.9%. Based on their findings, they recommend that an integrated bisulfite conversion efficiency control should be integrated in the protocol of studies examining methylation markers in cfDNA.

None of the included studies in this review had a description of DNA recovery and bisulfite conversion efficiency in their methods, nor mentioned the bisulfite treatment as a crucial step in their discussion.

## Conclusion

None of the examined genes found in this literature review are applicable as single diagnostic markers for HNSCC, and the best gene-panels still lack diagnostic accuracy. All the included studies evaluated predefined candidate genes based on earlier studies, and thus, future studies evaluating diagnostic methylation-markers in HNSCC may benefit from the use of whole genome sequencing, new microarray technology, and improved bioinformatics algorithms. Methylation levels seem to correlate with tumor size, stage, and nodal involvement so the diagnostic challenge of HNSCC may remain in small tumors with none- or uncharacteristic symptoms, but new panels with refined computational biology may be able to overcome this challenge.

Future evaluation of cfDNA methylation in peripheral blood could have a diagnostic as well as prognostic value and could be used for post-treatment surveillance. This would potentially improve the survival rate and reduce the treatment related morbidity in these patients by enabling early detection and less invasive procedures in the post-treatment follow-up care.

In conclusion, further studies are needed that ideally include thorough documentation of laboratory methods used and genome-wide analyses of single anatomical sites. This also includes analysis of correlation between methylation in tumor and peripheral blood, the HPV status, matched cases and controls, and sample size calculations. Altogether, these aspects may contribute to an improvement of HNSCC treatment regarding survival and the overall quality of life for these patients.

## Supporting information

S1 Checklist PRISMA 2009 checklist. (DOC)

S1 Table Search algorithms in PubMed and OVID EMBASE. (DOCX)
