# Supplementary material for: DNA methylation biomarkers in peripheral blood of patients with head and neck squamous cell carcinomas. A systematic review
Source: PLoS One. 2020 Dec 17;15(12):e0244101. doi: 10.1371/journal.pone.0244101 (PMC7746174; doi:10.1371/journal.pone.0244101)
Supplement: S1 Table — (DOCX) [file pone.0244101.s002.docx]

**S1 Table. Search algorithms in PubMed and OVID EMBASE**

**Search algorithm in PubMed**

1325 references

| #4 | #1 and #2 and #3 |
| --- | --- |
| #3 | "Carcinoma, Squamous Cell"[Mesh:noexp] OR "Squamous Cell Carcinoma of Head and Neck"[Mesh] OR Squamous Cell Carcinoma*[tw] OR Squamous Carcinoma*[tw] OR Planocellular Carcinoma*[tw]  AND head[tw] OR neck[tw] OR oral[tw] OR tongue[tw] OR larynx[tw] OR pharynx[tw] OR oropharynx[tw] OR nasopharynx[tw] OR hypopharynx[tw] |
| #2 | head[tw] OR neck[tw] OR oral[tw] OR tongue[tw] OR larynx[tw] OR pharynx[tw] OR oropharynx[tw] OR nasopharynx[tw] OR hypopharynx[tw] |
| #1 | "Methylation"[Mesh] OR Methylat*[tw] OR hypermethylat*[tw] OR hypomethylat*[tw] OR "Epigenesis, Genetic"[Mesh] OR Genetic Epigenes*[tw] OR Epigenetic Process*[tw] |

**Search algorithm in OVID EMBASE**

1103 references

| #16 | #5 AND #15 |
| --- | --- |
| #15 | #6 OR #7 OR #8 OR #9 OR #10 OR #11 OR #12 OR #13 OR #14 |
| #14 | hypomethylat*:ti,ab,kw |
| #13 | hypermethylat*:ti,ab,kw |
| #12 | methylat*:ti,ab,kw |
| #11 | 'epigenetic process*':ti,ab,kw |
| #10 | 'genetic epigenes*':ti,ab,kw |
| #9 | 'genetic epigenesis'/exp |
| #8 | 'hypomethylation'/exp |
| #7 | 'hypermethylation'/exp |
| #6 | 'methylation'/exp |
| #5 | #1 OR #4 |
| #4 | #2 AND #3 |
| #3 | head:ti,ab,kw OR neck:ti,ab,kw OR oral:ti,ab,kw OR tongue:ti,ab,kw OR larynx:ti,ab,kw OR pharynx:ti,ab,kw OR oropharynx:ti,ab,kw OR nasopharynx:ti,ab,kw OR hypopharynx:ti,ab,kw |
| #2 | 'squamous cell carcinoma*':ti,ab,kw OR 'squamous carcinoma*':ti,ab,kw OR 'planocellular carcinoma*':ti,ab,kw |
| #1 | 'head and neck squamous cell carcinoma'/exp |
